# Microalgae-Derived Pigments for the Food Industry

**DOI:** 10.3390/md21020082

**Published:** 2023-01-25

**Authors:** Han Sun, Yuxin Wang, Yongjin He, Bin Liu, Haijin Mou, Feng Chen, Shufang Yang

**Affiliations:** 1 Shenzhen Key Laboratory of Marine Microbiome Engineering, Institute for Advanced Study, Shenzhen University, Shenzhen 518060, China; 2 Institute for Carbon Neutrality, Shenzhen University, Shenzhen 518060, China; 3 Institute for Innovative Development of Food Industry, Shenzhen University, Shenzhen 518060, China; 4 College of Food Science and Engineering, Ocean University of China, Qingdao 266003, China; 5 College of Life Science, Fujian Normal University, Fuzhou 350117, China

**Keywords:** microalgae, carotenoids, phycobiliproteins, food colorant, stability

## Abstract

In the food industry, manufacturers and customers have paid more attention to natural pigments instead of the synthetic counterparts for their excellent coloring ability and healthy properties. Microalgae are proven as one of the major photosynthesizers of naturally derived commercial pigments, gaining higher value in the global food pigment market. Microalgae-derived pigments, especially chlorophylls, carotenoids and phycobiliproteins, have unique colors and molecular structures, respectively, and show different physiological activities and health effects in the human body. This review provides recent updates on characteristics, application fields, stability in production and extraction processes of chlorophylls, carotenoids and phycobiliproteins to standardize and analyze their commercial production from microalgae. Potential food commodities for the pigment as eco-friendly colorants, nutraceuticals, and antioxidants are summarized for the target products. Then, recent cultivation strategies, metabolic and genomic designs are presented for high pigment productivity. Technical bottlenecks of downstream processing are discussed for improved stability and bioaccessibility during production. The production strategies of microalgal pigments have been exploited to varying degrees, with some already being applied at scale while others remain at the laboratory level. Finally, some factors affecting their global market value and future prospects are proposed. The microalgae-derived pigments have great potential in the food industry due to their high nutritional value and competitive production cost.

## 1. Introduction

Presently, as consumers pay more attention to healthy diets and food safety, they are inclined towards the nutritive, natural and clean-label food products [1]. Following this proclivity, food and nutraceutical manufacturers are pursuing the exploitation and industrial production of natural high-value compounds. For pigments in food and health applications, a distinct move of market interest has also been driven towards biologically synthesized natural ingredients. Up to now, most of the commercially available food pigments are obtained through chemical synthesis and are generally considered not completely safe, or even toxic, owing to their molecular configurations or the organic reactions during their processing. Compared with synthetic counterparts, natural pigments have no toxic or side effects on the human body, and even act as nutritional enhancers with various biological activities. Although some plant-derived pigments have been commercially available, it has been found that the activity of microalgal pigments increases by multiple times. As a significant food ingredient, microalgal pigments are also chased by manufacturers and customers. The market value of microalgal pigments has been predicted to be USD 452.4 million by 2025 with a 4% Compound Annual Growth Rate (CAGR), and the market of microalgae products will be a large-scale business until that time [2,3].

Microalgae represent the important sources of value-added products, including proteins, lipids, polysaccharides, minerals, vitamins, pigments, and polyunsaturated fatty acids (PUFAs), which have great trading and health value [4]. Notably, “microalgae” themselves contain eukaryotic photosynthetic microorganisms along with prokaryotic cyanobacteria, which are responsible for converting light energy to chemical energy via photosynthesis. The three major classes of pigments, carotenoids (usually 0.1–0.2% of dry weight, DW or as high as 14% within certain species), chlorophylls (0.5–1.0% of DW) and phycobiliproteins (PBPs) (8% of DW) in microalgae are responsible for their photosynthetic pathways and cell growth. Compared with additional natural sources of pigments (such as fruits, plants, or animals), microalgae-derived pigments have been proved to contain outstanding physiological activities (such as antioxidant and antibacterial) and a wide spectrum of health applications. Various options of striking shades, hues and natural tones in the foods can be exhibited by microalgal pigments, thus endowing them with strong competitiveness as edible colorants that can mimic the color of natural food [5]. Furthermore, their biggest advantage lies in the culture characteristics of the microalgae themselves. 

As a stable natural source of pigments, microalgae can be cultured sustainably, in an eco-friendly manner, and are renewable on an industrial scale without the restriction of seasonal, climatic and environmental conditions. They are also outstanding for their high pigment content, fast growth rate, ability to grow in stress conditions, and the fact that they do not require arable land, thus they are becoming one of the most promising and competitive sources of natural pigments [6]. Some cultivation strategies are explored for enhancing pigment accumulation in diverse organisms. Some active pigments including PBPs (blue pigment extracted in *Spirulina*), astaxanthin (yellow-to-red pigment extracted in *Haematococcus*) and β-carotene (yellow pigment extracted in *Dunaliella*) have been scaled into industrial production and extensively used in food, nutraceutical, pharmaceutical, aquaculture, cosmetic and various industries. In microalgae, pigments are synthesized during vegetative growth or in stress conditions, and different biotic and abiotic factors in this process can affect the final quality of products [7]. The emergence of novel trophic modes and techniques effectively enhances pigment-producing efficiency [8]. Moreover, during the steps of extraction, purification and food processing, the structure of pigments may also be damaged or destroyed, thus affecting its coloring and nutritional function in foods. Therefore, it is essential to optimize the extraction and process methods to protect pigment stability and activity. 

This paper integrates the characteristics and distribution of three commercial microalgal pigments as chlorophylls, carotenoids and PBPs. Their current application patterns and functions in the food industry are reviewed. For the large-scale culture of microalgae, the influencing mechanisms of several environmental conditions on the final pigment yield was analyzed. In addition, the features and drawbacks of several conventional methods for pigment extraction are discussed. Finally, the current market value and future prospects are analyzed.

## 2. Chemistry and Biochemistry of Microalgal Pigments

**Figure 1:**
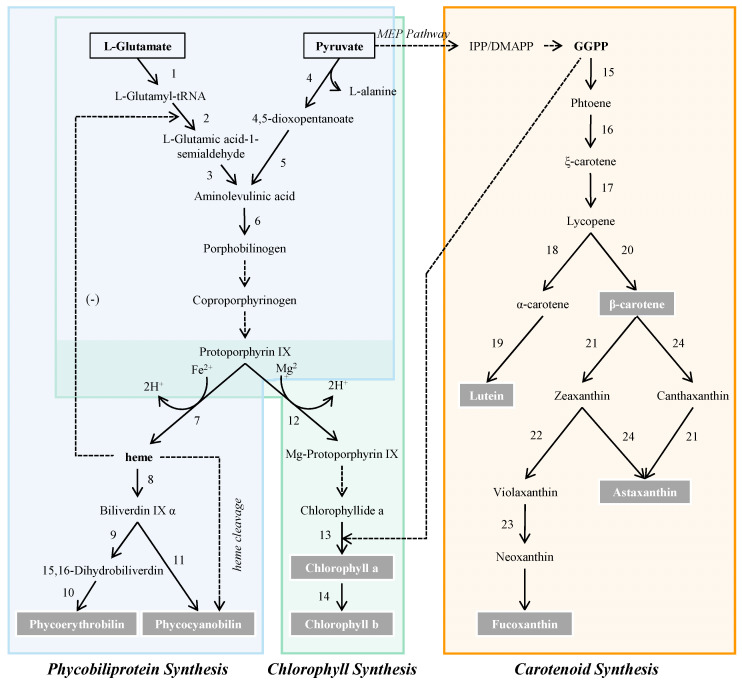
Biosynthetic pathway of pigments in microalgae and enzymes involved in pigment biosynthesis. 1, glutamate-tRNA ligase; 2, glutamate-tRNA reductase; 3, 5-aminolevulinate synthase; 4, 5-aminolevulinas; 5, aminolevulinic acid transaminase; 6, porphobilinogen synthase; 7, ferrochelatase; 8, heme oxgenase; 9, ferrodoxin oxidoreductase (Peb A); 10, ferrodoxin oxidoreductase (Peb B); 11, phycocyanobilin synthase; 12, Mg-protoporphyrinogen IX chelatase; 13, chlorophyll synthetase; 14, chlorophyll a oxygenase (chlorophyll b synthase); 15, phytoene synthase; 16, phytoene desaturase; 17, zeta-carotene isomerase, zeta-carotene desaturase, carotenoid isomerase; 18, lycopene epsilon cyclase; 19, cytochrome P450-β hydroxylase; 20, lycopene-β cyclase; 21, β-carotene hydroxylase; 22, zeaxanthin epoxidase; 23, neoxanthin synthase; 24, β-carotene ketolase. Abbreviations: IPP, isopentenyl pyrophosphate; DMAPP, dimethylallyl pyrophosphate; GGPP, geranylgeranyl pyrophosphate.

Microalgae represent the important natural pigment sources, which play an irreplaceable role in photosynthetic carbon fixation and cell growth [4]. In addition, the type and quantity of natural pigments in microalgae varied with the species (Table 1). The common biosynthetic pathway of chlorophyll, phycobiliprotein and carotenoid was shown in Figure 1. With unique structure and various beneficial functions, these pigments have been explored and utilized to different degrees in a wide spectrum of fields.

Chlorophylls are greenish pigments discovered in oxygenic photosynthetic organisms including plants, microalgae and cyanobacteria. They are responsible for photosynthesis by absorbing and converting solar energy into chemical energy. At present, five categories of chlorophylls have been identified, including a–f, and their maximal absorption wavelengths (λ_Amax_) are, 665, 652, 630, 696 and 707 nm, respectively. Most pigments can bind to several phyla, while chlorophyll a is the most abundant in microalgae [25]. Chlorophyll b represents the pigment with the second highest abundance within green microalgae; on the other hand, chlorophyll c has been observed within haptophytes, dinoflagellates, cryptophytes and heterokonts; while chlorophyll d and f were identified in Rhodophyta, cyanobacteria, and dinoflagellates. On average, microalgae contain 0.5–1.0% of chlorophylls per DW [26]. Many microalgae can be the source of natural chlorophylls used for commercial applications, such as *Chlorella* sp., *Monoraphidium dybowskii*, *Scenedesmus dimorphus*, *Chlamydomonas reinhardtii*, *Pavlova lutheri*, *Ankistrodesmus falcatus*, and *Chlorella vulgaris* [27].

Carotenoids can be dissolved in lipid, and their colors can be red, brown, yellow or orange. Carotenoids include the terpenoid pigments that can be obtained in the 40-carbon polyene chain and end with “ionone” rings. The different architectures provide carotenoids with outstanding light-absorption characteristics necessary in the photosynthetic process. On the basis of chemical structure, these hydrocarbons are classified into carotenes and xanthophylls. Carotenes, namely hydrocarbons, consist of α- and β-carotene. Xanthophylls contain oxygen as hydroxyl groups (such as zeaxanthin, lutein), keto-groups (such as echinenone, cantaxanthin), or their combination for violaxanthin and astaxanthin. Carotenoids are divided into primary or secondary ones. The primary ones act as the cellular photosynthetic apparatus’ functional components, which are linked with membranes or specific proteins in the thylakoid membrane (e.g., xanthophylls), and the secondary carotenoids are typically produced when exposed to specific environmental stimuli and exist in lipid vesicles. Carotenoids have two important effects on photosynthesis: (1) light absorption within visible spectral regions where no chlorophyll is effectively absorbed; (2) photoprotection on photosynthetic systems. Typically, the photoprotective mechanisms have been proven to weaken the energetic state change in chlorophyll induced by excess light radiation absorption. It can effectively hinder reactive oxygen species (ROS) production and endows carotenoids with superior antioxidant capacity [28]. Over 600 carotenoids have been identified, while those in microalgae are mainly lycopene, β-carotene, zeaxanthin, astaxanthin, lutein and violaxanthin, which nearly compose 90% of carotenoids within the human body and the diet. Among them, β-carotene, astaxanthin, fucoxanthin and lutein are the most studied ones.

β-carotene is the vitamin A precursor (retinol), which represents an orange-yellow color depending on the quantity of β-carotene and other pigments. Demand for natural β-carotene is increasing, for this carotenoid presents more superior bioactive properties than artificial ones. The microalgae *Dunaliella* contains β-carotene above 14% DW and has long been recognized as the largest natural source [26]. *Scenedesmus quadricauda* produces 19 mg g^−1^ β-carotene under autotrophic cultivation [29]. Other microalgae of Cyanobacteria, Chlorophyta, Bacillariophyta, and Euglenophyta also master the capability to produce β-carotene. Astaxanthin is a byproduct of β-carotene with a rosy color, it is converted from β-carotene under the action of β-carotene hydroxylase (CRTR-B) and β-carotene oxygenase (CRTO) under stress, followed by accumulation within lipid vesicles out of chloroplasts. In nature, astaxanthin usually exists with 1 or 2 esterified fatty acids (FAs, referred to as monoester and diester, respectively). Astaxanthin can form as different isomers according to hydroxyl group configuration: 3S and 3’S, 3R and 3’S (meso), and 3R and 3’R. Microalgae including *Haematococcus pluvialis*, *Chlorococcum* sp. and *Chlorella zofingiensis* have a capability to accumulate astaxanthin in vesicles when subjected to stress [30]. Lutein is a lipophilic tetraterpene, whose color ranges from yellow (low content) to red-orange (high content). The double bonds in lutein endow its high antioxidant activity to be chemically reactive with ROS. On the other hand, lutein can protect photoreceptors by filtering blue light (500 nm) [31]. One of the key commercial sources of lutein production is *Muriellopsis* sp., which contains lutein at 0.4–0.6% per dry biomass [32]. *C. zofingiensis, Chlorella protothecoides,* and *Scenedesmus* spp. have also been studied for lutein production. Until now, just *Scenedesmus almeriensis* and *Muriellopsis* sp. realized the mass production, which are cultured massively within the outdoor systems with continuous and feasible operation [33]. The orange pigment fucoxanthin accounts for more than 10% of total natural carotenoids and is ubiquitous among living organisms in a marine ecosystem. Fucoxanthin has a multi-olefin skeleton and specific conjugated double bonds, as well as single epoxy, carbonyl, and hydroxyl groups [34]. These unique bioactive structures in fucoxanthin are responsible for its potent health benefits to humans, such as anti-obesity, anti-cancer, and blood glucose regulatory functions. Diatoms (*Bacillariophyta*, especially *Odontella* sp.) and brown microalgae (*Phaeophyceae*) have abundant levels of lutein, but their application in industrialized production is still not feasible. *Isochrysis galbana* can generate 1.2–15-fold increased fucoxanthin compared with the conventional macroalgal source [35]. *Tisochrysis lutea* is an alternative source for this pigment, 79 mg g^−1^ of fucoxanthin can be extracted from its mixotrophic culture [36]. Some other microalgae, such as *Phaeodactylum tricornutum*, *Cylindrotheca* sp., *Odontella aurita*, *Chaetoceros muelleri*, *Amphora* sp., *Navicula* sp., and *Chrysotila carterae*, also showed considerable fucoxanthin production capacity [37,38].

PBPs are soluble in water and are relatively easy to isolate and purify. They can generally be divided into phycoerythrin (PE, red, absorption detected at 565–575 nm), phycocyanin (PC, blue, absorption detected at 651–655 nm), as well as allophycocyanin (APC, green-blue, absorption detected at 650–655 nm) in line with spectroscopy characteristics and amino acid sequences [39]. PBPs, as oligomeric proteins, are comprised by chromophore-bearing polypeptides. They can be assembled into phycobilisomes or adhered onto thylakoid membrane for photosynthesis. In some microalgae that are lacking chlorophyll b, PBPs are synthesized for compensating for the huge chlorophyll a absorption gap while optimizing light energy collection. In the photosynthetic process, such PBPs can capture light energy, subsequently passing it into the chlorophylls. PBPs have been widely used as food additives and/or excellent nutrient supplements for the human diet, demonstrating a number of healthy functions. In most cyanobacteria, cryptophytes, glaucophytes and red algae, the common PBP is PC, which is especially rich in numerous cyanobacterial strains under the natural environments. Nowadays, PC can be generated from autotrophic cyanobacteria *Spirulina platensis* because it is ubiquitous. A strain of *Geitlerinema* can also achieve a yield of 172 mg g^−1^ [40]. PE is a kind of PBP with the highest abundance level within numerous red algae as well as certain cyanobacteria, it can now be produced from *Porphyridium* sp. (such as *P. purpureum* and *P. cruentum*), and its content is 15% DW (as high as 30% in the ideal environment), while its yield is 200 mg L^−1^ [41]. In comparison to PC and PE, the massive commercial APC manufacturing process has not been established so far because it is limited by the poor intracellular pigment content and biomass generation.

Several unusual minor pigments exist in microalgae, including prasinoxanthin, loroxanthin, siphonaxanthin, peridinin, flavonoids, and tannin [27]. Their structural variety and beneficial activities are attractive in the food and pharmaceutical industries. The pigment analysis of seven prasinophyceans (Chlorophyta) distinguished three groups that contained prasinoxanthin, loroxanthin ester and siphonaxanthin, respectively [42]. The bioactive compound analysis revealed that *Nannochloropsis* sp. contained 7.25% β-sitosterol, and 10.3% stigmasterol [43]. As little information is available for the biotransformation of such unusual pigments during the metabolic process, further studies are required to reveal the bioavailability and biodigestibility of these pigments.

## 3. Potential Application of Microalgal Pigments and Target Foods

**Figure 2:**
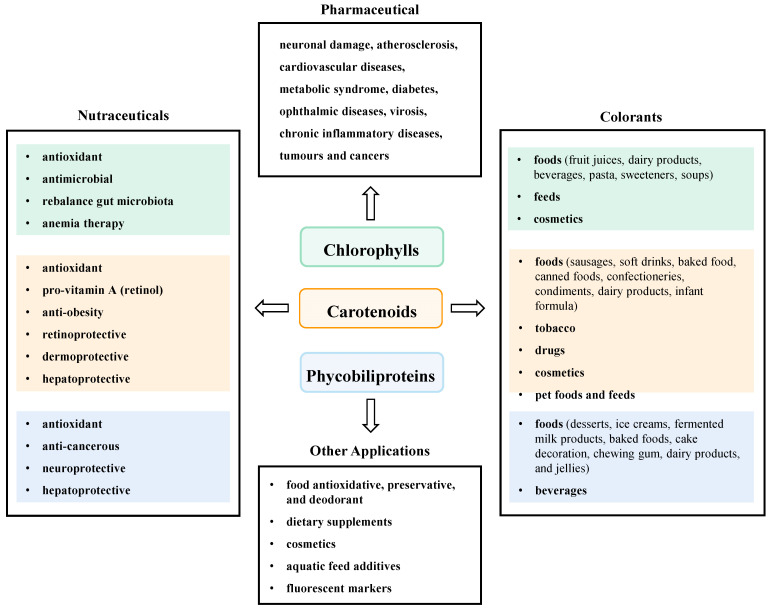
Application of microalgal pigment in various fields.

With unique molecular structure and various beneficial activities, microalgal pigments have been reckoned as promising eco-friendly colorants, nutraceuticals and antioxidants with great commercial value in food and dairy, alternative foods, dietary supplements, pharmaceuticals, cosmetics, aquaculture, textiles, and other light manufacturing fields [44,45]. At present, these pigments have been developed and utilized to different degrees, some of them have achieved efficient industrial production and high economic value. This section reviewed the popular application examples and acting modes of microalgal pigments in food and other industries (Figure 2).

### 3.1. Chlorophylls

Similar to most natural pigments, chlorophylls have numerous functions in food additive industry, within which, the most extensive application is colorants. Out of the antipathy to synthesized pigments with potential side effects, food industries now tend to use natural green colorants to endow, intensify, or uniformize color of foods, thus presenting best color intensity and hue to satisfy consumers’ expectations, requirements and needs [46]. At present, microalgae have been identified to be the sustainable source to produce natural food-grade colorants with low allergenicity, toxicity and carcinogenicity. The common application of microalgal pigments including chlorophylls and their target food products as colorants is listed in Table 2. As the most abundant natural pigment with green color that are rare in foods, chlorophylls are currently gaining immense attention as food, feed, cosmetic and pharmaceutical colorants and functional food supplements [47]. Addition to colorants, such as microalgal pigments can display auxiliary effects of preservation and antisepsis in food storage, as the consequence of their physiological activities. Chlorophylls were proven to serve as an excellent deodorant of foods [48].

Microalgal pigments can also be utilized in nutraceuticals and as dietary supplements. Due to their excellent bioactive properties, chlorophylls have been consumed as nutraceutical agents and antioxidants. They can also contribute to rebalancing the gut microbiota and decreasing colorectal cancer risk. Since chlorophylls regenerate or act as substitutions for hemoglobin in deficiency conditions, the utilization of chlorophylls supplement has been recommended for thalassemia and hemolytic anemia adjuvant therapy [49]. Chlorophyll compounds are also suggested to have medicinal application with wound-healing, antimicrobial, anticancer, antimutagenic, antitumor and anti-inflammatory properties [50,51]. Furthermore, they may be applied to be components of dentifrice or in producing cosmetics for skin care. Chlorophyllin represents the chlorophyll derivative, where copper or sodium replaces magnesium, with the loss of phytol chains. It is employed as a dietary supplement and was found to exhibit antimutagenic and anticarcinogenic action.

### 3.2. Carotenoids

With striking color characteristics and bioactivity, natural carotenoids are the best colorant option for food producers, particularly for cooked sausages, soft drinks, and baked goods. Apart from that, its excellent antioxidative and preservative attributes help food retain original color and taste during storage, and preventing FAs and other substances from oxidizing and deteriorating [52,53]. Thus, they have recognizable impact and demand in foods with increased FAs (such as butter, margarine, milk products, soft drinks and cakes). 

Carotenoids also have the effects of antioxidation, anti-inflammation, neuroprotection, which can also improve age-related macular degeneration and cardiac dysfunction. Carotenoids can effectively hinder the formation of intracellular ROS and play a strong physiological antioxidant effect [28]. Microalgal carotenoids contain trans- and cis-enantiomers that further enhance the bio-efficacy and bioavailability. They are also bioactive and can resist cancer because they contain xanthophylls. Thus, these biomolecules have been applicated into numerous fields, including cosmetics, pharmaceutical, functional foods, nutraceuticals, agriculture and aquatic feeds [54]. 

β-carotene represents the red pigment, which is currently most popular and has been widely applied to many food products (e.g., cakes, margarine, butter, cheese, canned foods, baked products, health condiments, confectioneries, dairy products and soft drinks) and pet foods to enhance their attractiveness. Since vitamin A enhances body immunity while preventing cataracts, cutaneous disorders and night blindness, it is reckoned as being a necessary nutrient for the human body. β-carotene can be adopted to be the pro-vitamin A (retinol) in multi-vitamin preparations, which is also used to formulate cosmetics (such as sunscreen formulations) or healthy foods. β-carotene can also act as a strong antioxidative agent (healthy food or cosmetics additives), a hepatoprotective agent, a cosmetic or a multi-vitamin preparation additive, and as a functional food component [55,56]. As a health care product, it offers medical effects that can be described as being retinoprotective, dermoprotective, anti-inflammatory, antihypertensive, antitumor, and antidiabetic and these effects cannot be achieved by a synthetic version [57]. Additionally, it can be applied in animal feed for improving fish (such as salmon), crustaceans, and a bird’s appearance (color). 

Astaxanthin is also a carotenoid with important commercial value, given it is a natural colorant. Astaxanthin extracted from *H. pluvialis* is approved in Japan, the USA as well as some European countries to be used in human dietary supplements and salmonid feeds. Moreover, bread that contains *H. pluvialis*-extracted astaxanthin is also developed by researchers, which is under favorable preservation against degradation in the process of baking [58]. Astaxanthin is a superb antioxidant with a 12-fold increased activity compared with vitamin E by sequestering free radicals and quenching ROS. There is also an increasing market interest of its anti-obese activity, which now has applications in nutraceuticals, dietary supplements, cosmetics, pharmaceuticals, and the healthcare area [59]. An astaxanthin-containing oral preparation was already developed to treat Helicobacter infections in the gastrointestinal tract (GIT). The experiments revealed the great capability of natural astaxanthin to act as hepatoprotector, anticancerogen and anti-aging compound [60]. It can be used in the adjuvant treatment of neuronal damage, central nervous system injuries, heart disease, atherosclerosis, diabetes, chronic inflammatory disorders, cutaneous disorders and in functional foods. 

Lutein has been considered as a significant nutraceutical, which can be applied in food, cosmetics and drug coloration. It is now commercially added to flavorings, tobacco, pastries, confectionery, infant formula and a variety of feed products to provide them with a distinctive and attractive color. When used as a nutraceutical, lutein generally has effects on eye structure and vision-related conditions (macular degeneration, retinopathies or cataracts associated with age). Since blue light radiation has unfavorable effects on human eye macula, lutein protects retina through filtering blue light (500 nm) and reducing 40% of light incidence [31]. Moreover, investigations of lutein for its anti-inflammation and antioxidation effects go beyond these eye diseases. Lutein has been successfully applied in nutraceuticals for the prevention of other chronic disorders such as cardiovascular diseases (CVDs), diabetes, atherosclerosis, and together with some cancers, have gained widespread attention associated with human health [61]. As revealed by some studies, lutein protects against solar UV radiation-induced skin damage, thus displaying its potential application in skin care products such as sunscreens.

Microalgal fucoxanthin production has not achieved industrialization on a large scale, and its application as a food colorant is limited. It can be applied to egg yolk, butter, pie, green tea cakes, baked foods and dairy products for a better color and functional role [62]. Fucoxanthin from *P. tricornutum* added to dairy products was proven to improve its stability and bioavailability. Natural fucoxanthin from diatoms obtains a great potential for application on cosmetics, pharmaceuticals, food, poultry feed, and aquaculture industries, due to its unique bioactive structures and potent health benefits to humans. Studies have demonstrated that consuming fucoxanthin in dietary supplements and beauty products can serve as an antioxidant and scavenger for ROS. For obese people, fucoxanthin has been proven to have outstanding weight loss benefits and can be used in weight loss supplements in diets. In the medical field, it also helps to cur many chronic diseases such as type 2 diabetes (T2DM), heart disease, high cholesterol, osteoporosis, hypertension, metabolic syndrome, liver disease, and some specified cancer (e.g., skin, colon, prostate, liver cancer and other cancers). Notably, the combination with edible lipids can increase the rate of fucoxanthin absorption when utilized as a nutritional supplement [57]. Fucoxanthinol, a deacetylated fucoxanthin, is extracted in diatom *Nitzschia laevis*, which shows neuroprotection; therefore, it can be used in food supplements and pharmaceutical products [63]. 

### 3.3. Phycobiliproteins

PBPs are widely used in the commercial sector as natural colorants. The red or pinkish-red PE as a food colorant has received several patents in foodstuffs, including confectioneries, syrups, dairy products, baked foods, gelatin desserts, dried foods, fermented milk products, ice cream and milk shakes [41,64]. In addition, PE has yellow fluorescence properties and can be added in cake decoration, soft drinks, and alcoholic beverages to endow them with fluorescence properties and improve their appeal to consumers [41]. As to its natural blue color, PC from cyanobacteria is being adopted to color ice cream, soft drinks and yogurt in Japan. It can also be added in chewing gum, candies, popsicles, jellies, dairy products and soft drinks. For example, a PC-enriched yogurt showed positive effects in coloring stability during storage [65]. The natural protein pigment C-phycocyanin (C-PC) in *Spirulina* is verified with high safety, which can be used in beverages and foods as a colorant [66].

PBPs have antioxidation, anti-inflammation and liver protection effects, thus giving them outstanding potential in human health. PC is reckoned as a strong in vivo antioxidant with radical scavenging properties. In experiments, PC can act as a functional food with excellent anti-inflammatory, anti-cancerous, auxiliary hepatoprotective and neuroprotective effects. APC is reported to hinder virus-induced entero cytopathic impacts, viral plaque formation as well as viral-induced apoptosis, while C-PC shows strong hypocholesterolemic activity by adjusting serum cholesterol concentrations. Some studies have shown that PE is a novel photosensitive drug with high efficiency and no side effects, which can be applied to kill tumor cells. 

Due to their bioactive properties, PBPs are also extensively utilized in immunological laboratories and industries. Generally, they have been usually used as the fluorescent markers in molecular biology and as fluorescent dyes in microscopy and immunoassays [26]. Over 297 patent records are available for PBPs such as fluorescent markers. For example, PC and PE collected in the cyanobacterial species have been adopted to be fluorescence probes in gel electrophoresis as the protein markers.

## 4. Factors Affecting the Microalgal Pigment Production

Nowadays, fermentation of microalgae has become one of the effective strategies of natural pigment production. Compared with those from plants and aquatic animals, pigments obtained from the industrial production of microalgae have a multitude of advantages, including controllable production, easy extraction, high yields, no raw materials scarcity, and no seasonal variations. Within the culture period, any slight change of environmental conditions can cause an alteration of the pigment productivity and molecular structure, ultimately affecting the market acceptance, and bioaccessibility of final products.

### 4.1. Light

Light is the most affecting factor of converting inorganic carbon into organic molecules in phototrophic organisms. Light utilization can proportionally promote microalgal growth, which is under the regulation of accurate light intensity and photoperiod [67]. Light intensity represents the most obvious and easily regulated factor that affects cell photosynthesis and pigment biosynthesis. Light has a negative effect on photosynthesis under extreme light conditions out of its tolerance limit through the destruction of photosynthetic apparatus. Phycobiliprotein and chlorophyll production is the light collection-related adaptive response. Low light intensity leads to the decreased specific maintenance energy ratio in cyanobacteria, which also stimulates its phycobiliprotein production. 

Experiments showed that 25–50 μmol photons/m^2^/s was the highest phycobiliprotein generation intensity in some blue-green microalgae, while that of *Spirulina* increased with the increases in light intensity (135 μmol photons/m^2^/s) [68]. The chlorophyll content in several microalgae (including *Chlorella* sp., *Dunaliella salina*, *C. reinhardtii*, and *S. platensis*) displays an inverse relationship to light intensity [69]. In contrast, elevating light intensity shows a positive effect on β-carotene synthesis and accumulation of microalgal cells. *D. salina* was validated to have an enhanced β-carotene productivity at higher irradiation [70]. Large-scale cultivation of microalgal carotenoid (such as lutein and astaxanthin) mostly operates in two steps, cultivating microalgae in optimum conditions for fast growth and then improving desired carotenoid content in stress conditions [71]. Astaxanthin accumulation in *H. pluvialis* was reported to be enhanced (30 mg/g) as the result of enhanced oxidative stress under intense illumination (>500 μmol photons/m^2^/s) [72]. For lutein production, *Tetraselmis* sp., *Scenedesmus* sp., and *Chlamydomonas* sp., respectively, gain highest productivity at 170, 300 and 625 μmol photons/m^2^/sec light intensities [73,74,75].

The significance of the light quality on photosynthetic pigments goes beyond that of the light intensity under certain circumstances, and even influences cell maturity, culture density, light path and the medium nutrients profile. Discontinuous illuminating strategies such as the light/dark photoperiod cycle and the flashing light effect have been used to achieve higher light availability. The photoperiod effectively regulates microalgal chlorophyll levels [76], and usage of the flashing light effect in industrial culture can enhance the astaxanthin generation rate 4-fold within *H. pluvialis* per photon relative to continuous light sources [77].

Light color is also related to pigment generation within diverse microalgae. The photoreception systems’ absorption and utilization of light depend on wavelength of the incident light to a great extent. In order to ensure the highest light availability, the most suitable light wavelength should be chosen for target pigment production. In some studies, red light is found to favorably enhance PBPs generation within most blue-green microalgae, whereas blue light stimulated that of *Spirulina* sp. and enhanced its chlorophyll production. Green light increases the C-phycoerythrin (C-PE) level in nitrogen-fixation cyanobacteria, and red light irradiation increases the C-PC level [78]. The intensity of white light is helpful for synthesizing chlorophyll as well as accumulating C-PC in *Spirulina* sp., whereas green light positively affects its PC generation [68]. The optimal lutein production of marine microalga *Chlamydomonas* sp. can be achieved in blue light at 20–25 °C [74].

Light spectrum is the photomorphogenic signal in microalgae cultures. Various lengths of spectrum, including blue: red, red: far red, green: red, and blue: green have been often used at different proportions to achieve the best induction effect. Alteration of spectrum proportions influence the relative pigment composition. The lutein production from *Scenedesmus obliquus* could obtain the maximal productivity (1.43 mg L^−1^ day^−1^) with 4-day blue followed by 4-day red exposure [79]. Chlorophyll, a content of *Chlorella pyrenoidosa,* declines after blue light irradiation, while it greatly elevates after blue: red irradiation [80]. Blue: green lights are helpful for high *Pyropia haitanensis* effectiveness and increased pigment levels [81]. *G. membranacea* and *C. vulgaris* attain highest chlorophyll a levels after red: green light irradiation [82].

### 4.2. Temperature

Temperature is the elementary factor that can govern the rate of all metabolic processes and cellular component structure by influencing membrane fluidity, enzymatic activities, as well as electron transport chain efficiency. The suitable growth temperature and extreme value tolerance are usually different among diverse microalgal strains.

Typically, high temperatures can promote microalgal pigment production. A range of 25–28 °C was reported as optimum in terms of chlorophylls accumulation temperatures, for higher temperatures may stimulate damaging of cells by osmotic pressure [69]. Likewise, 28 °C is the best temperature of astaxanthin generation in *H. pluvialis* [83], and 30 °C is optimum for *C. zofingiensis* [84]. Blue-green microalgae can generate numerous carotenoids at high temperature, in particular β-carotene [27]. Total carotenoid production from microalgae *H. pluvialis* and *Phormidium autumnale* have demonstrated the maximum yield, respectively, at 23 °C and 26 °C [85,86]. For D. salina, a temperature at 30 °C leads to its highest β-carotene production [87]. *Muriellopsis* sp., *C. protothecoides*, *C. zofingiensis*, and *Neospongiococcus gelatinosum* display best lutein productivity when cultured at 28 °C [88]. In case of PBP, the optimum temperature for its production is at 25 °C, 30 °C, 35 °C and 36 °C for *S. platensis*, *Anabaena* sp., *Nostoc* sp., as well as *Synechococcus* sp. [78,89]. 

### 4.3. Culture Media

#### 4.3.1. Nitrogen

Nitrogen is the principal nutrient requirement for microalgae growth and protein/chlorophyll molecule/nucleic acid production. Nitrogen deficiency has been identified for inducing various cell responses within microalgae, such as stimulating excessive free radical formation. Since carotenoids production has been recognized to be the protective response to photo-oxidative stress resulting from the excessively reduced photosynthetic electron transport chain, nitrogen deficiency can cause a marked increase of their contents. On the other hand, four nitrogen atoms are required to compose four pyrrole groups for the synthesis of each chlorophyll molecule and nitrogen stress displays an adverse effect on its synthesis. *C. vulgaris*, *Scenedesmus subspicatus*, *Chlorella fusca*, *S. platensis* and *C. reinhardtii* have a reduced chlorophyll level and growth dose-dependently as a consequence of nitrogen deficiency [90]. Cyanobacteria shows particular requirements for nitrogen sources, many blue-green microalgae (such as *Anabaena* sp.) were proven to produce a high amount of PBPs with nitrogen-free condition, while *Fischerella* sp. showed the opposite tendency [78].

#### 4.3.2. PH and Salinity

Little research is conducted to investigate how pH affects pigment production in microalga, but its alteration can effectively influence nutrient bioavailability and solubility in a culture system. pH levels < 5.0 and >8.5 are proven to suppress microalgae growth [67]. pH confines the substance dissolution and cell nutrient absorption. *S. platensis* generated the highest levels of C-PC (91 mg/g DW), carotenoids (2.4 mg/g DW) and chlorophyll a (10.6 mg/g DW) at pH = 8.5, whereas PC (159 mg/g) at pH = 9.0 [2]. A study of cyanobacterium *Nostoc* sp. showed that an increase in pH could result in elevated content of PC, PE and APC, and optimum total PBP production was found at pH 9. The maximum PBPs and chlorophylls production in blue-green microalgae (such as *S. platensis*) were respectively obtained at pH 8 and pH 9 [69,78]. As to the carotenoid, pH 7–8 is identified as the optimal pH value to generate carotenoids within most green microalgae, typically, the best pH to produce β-carotene is 7.5 for *D. salina*. However, such pH alterations within some microalgal culture mediums can suppress carotenoid and chlorophyll synthesis.

Salinity plays a vital role in industrial pigment production of both marine and limnetic microalgae, and osmosis shows a great impact on pigment accumulation. Rapid entry of sodium ions into cells results in phycobilisome detachment from the thylakoid membrane, leading to a significant reduction of photosynthesis and a restriction of pigment production. Thus, excessive salinity is usually not expected, for it produces hypertonic solution in the culture liquid and causes cell shrinkage. The chlorophylls and total carotenoids content decreases with the increase in salinity; their optimum productivities were found at low salinity (2–3 ppt) for most microalgae [70]. On the other hand, β-carotene generation increased within blue-green microalgae as salinity levels elevated. As a result, 10–15 ppt of salinity was proven to maximize the PBPs generation of blue-green microalgae *Anabaena* sp. (135.73 mg/g) and *Oscillatoria* sp. (66.7 mg/g) [78].

#### 4.3.3. Micronutrients

Micronutrients (such as manganese, iron and zinc) have critical effects for pigment metabolic pathways and generally low or even trace amounts are needed. Iron is involved in the tricarboxylic acid cycle and other metabolic pathways within *C. pyrenoidosa*, chlorophyll level decreased synchronizing with iron restriction. Iron electrical valency as well as counter ions affect astaxanthin accumulation [27]. Astaxanthin production can be enhanced after iron supplementation into the medium [85]. By adding 18 mM Fe^2+^-EDTA, astaxanthin synthesis can be effectively stimulated. β-carotene content also elevated significantly when supplied with 450 mM FeSO_4_ [91]. 

Copper serves as an essential cofactor for metalloenzymes in several metabolic pathways but is toxic for microalgae growth at high concentrations. A 1 mg/L copper cultures has been proved to hinder the growth of *I. galbana*, *Pavlova viridis*, and *P. tricornutum*, and meanwhile reduce their chlorophyll content. High zinc and copper contents are associated with chloroplast membrane peroxidation caused by free radical production, thus leading to reduction of chlorophyll content.

Magnesium ions, the pivotal chlorophyll ions, participate in pigment biosynthesis; moreover, they are also related to pigment metabolic pathway as the cofactor for critical enzymes [92]. Chlorophyll levels in cells of *Chlorella* sp. show a gradual decrease with and without magnesium restriction. 

Sulfur deprivation represents another modification within the microalgae culture environment. Microalgae require sulfur for producing numerous essential metabolites, such as sulfur-containing amino acids cysteine and methionine. Sulfur deprivation reduces oxygenic photosynthesis, promotes the activation of hydrogenase and has a negative effect on microalgal chlorophyll accumulation in *C. reinhardtii* and *C. fusca* [93,94]. On the contrary, sulfur deprivation displays a promoting effect for carotenoids biosynthesis. It is a more efficient approach to induce astaxanthin and lipids accumulation in *H. pluvialis* compared with nitrogen restriction, which was also verified in *C. reinhardtii* and *Parachlorella kessleri* [93,95].

## 5. Metabolic and Genomic Design for Pigment Production

The recent few years has seen an increased interest in the exploitation of microalgae as recombinant platforms for pigment production [96], requiring easy and efficient genetic engineering methods targeting nuclear and chloroplast genomes. 

Compared to plants, the research of microalgae transgenic system started late. Transformation of microalgae was first accomplished within *C. reinhardtii* by using the high-speed microprojectiles in 1988 and it was also the first report of chloroplast conversion [97]. Ever since then, great progress has been made on the transgenic approach of this species, and achievements has been applied to other algae species [98]. Apart from *C. reinhardtii*, tremendous advances are attained in genetically engineering other several microalgal species, such as *Nannochloropsis* sp. [99], *P. tricornutum* [100], *Chlorella* spp. [101] together with *C. vulgaris* [102]. The most frequently used method of transformation includes glass bead method, electroporation, agrobacterium-based transformation, PEG-mediated method and biolistic delivery. Apart from transformation methods, regulatory elements also affect the transformation efficiency and stability such as vector backbones, promoters, UTR, selectable and screenable markers [103]. Since carotenoid is primarily synthesized in the chloroplasts of microalgae, the transformation of either nuclear or chloroplast is available to improve the production. 

Sufficient isoprenoid precursor is the prerequisite for the massive carotenoid accumulation. Overexpressing the key enzymes involved in the synthetic pathway is the efficient strategy to increase carotenoid content. The phytoene synthase (PSY), phytoene desaturase (PDS) and beta-carotene ketolase (BKT) are reported as the key enzymes during carotenoid biosynthesis. The overexpression of *PSY* gene from *D. salina* in *C. reinhardtii* resulted in an improved carotenoid content, of which violaxanthin, lutein, neoxanthin and β-carotene showed a 2, 2.6, 1.8 and 1.25-fold increase compared with the wild type [104]. Introducing PSY into *P. tricornutum* at the exponential phase increased fucoxanthin content by about 1.45-time [105]. The internal PDS was codon optimized and overexpressed in *H. pluvialis*, and astaxanthin content was up to 67% higher than the wild-type (WT) [106]. The BKT and beta-carotene hydroxylase (CRTR-B) in *H. pluvialis* were introduced into *Dunaliella viridis* to produce astaxanthin and canthaxanthin content at 77.5 and 50.1 μg/g, respectively [107]. Alternatively, microRNA-mediated silencing was applied to knock down the autophagy-related genes *ATG1* and *ATG8*, which increased the β-carotene content at 2.34-fold of the wild type [108]. 

The carbon flux and available carbon molecule are anticipated to guide pigment biosynthesis for high carbon efficiency [109]. The carbon skeleton and energy for pigment biosynthesis are mainly from Calvin-Benson-Bassham (CBB) cycle as autotrophic mode or Embden-Meyerhof-Parnas (EMP) pathway as heterotrophic mode [110]. As the active photosystem was conducive to pigment accumulation, the low light and blue light was applied to elevate acetyl-CoA and pyruvate for improving fucoxanthin content in *I. zhangjiangensis* and *N. laevis* at 22 and 11.1 mg/g, respectively [111,112]. The red light was beneficial for cell division with high chlorophyll but the blue light was favorable for astaxanthin accumulation in *H. pluvialis* at 91.8 mg/L yield [113]. As the secondary metabolite, astaxanthin usually competes the carbon availability with protein accumulation for cell growth. The addition of glucose could vastly promote carbon availability in microalgae, but it restrains the photosystem, thus limiting the pigment biosynthesis. As nitrogen deficiency was an effective condition to up-regulate the genes involved in carotenoid biosynthesis several times, it was combined with carbon-based fed-batch culture to guide the elevated acetyl-CoA and pyruvate into astaxanthin biosynthesis in *C. zofingiensis* at 2.0 mg/L/d productivity [114]. The metabolic and genetic engineering approaches are efficient to improve pigment production from microalgae, but the food safety concerns and consumers’ acceptance are still required to be overcome.

## 6. Downstream Processing for the Stability of Microalgal Pigments

### 6.1. Pigment Stability by Extraction Technology

Pigments produced by microalgae fermentation demand effective technologies for cell (membranes and/or cell wall) disruption and extraction. The stability of target pigments is one of the most fundamental requirements during extraction, but is also easily destroyed by extreme conditions, impacting the final quality and bioaccessibility of products. For the extraction of microalgal pigments, appropriate methods should be chosen to protect their structure. Common extraction methods can be divided into mechanical (e.g., bead milling, pressure, ultrasonication, microwave, and electric fields), chemical (supercritical extraction) and biological (enzymatic) methods. Table 3 presents a summary of techniques mentioned in this section to obtain microalgal pigments.

#### 6.1.1. Classic Methods

Solvent extraction is a classic method for microalgal pigments [144]. A solvent can be absorbed within the cell wall, causing perforation or dissolution of the membrane and/or wall, and acting as the extractant of intracellular compounds. The selection of the most effective solvent relies on their chemical affinities for target pigments and the composition of a microalgal membrane or cell wall. Carotenoid extraction can be performed with non-polar solvents due to their high hydrophobicity. For instance, lutein extraction from wet *C. vulgaris* was investigated, with ethanol/hexane 3:1 (*v*/*v*) being proven to be the most suitable solvent [145]. Fucoxanthin from *I. galbana* can be intactly obtained via ethanol extraction [35], and astaxanthin extraction from *H. pluvialis* is performed using chloroform/methanol or acetone/ethyl acetate/ethanol [115]. Since natural pigments are thermally sensitive compounds, their solvent extraction should be operated at appropriate temperatures. While the application of thermal treatment can improve the extraction rate, excessive exposure to high temperatures (usually over 65 °C) leads to structure degradation, which has been verified on carotenoids and chlorophylls [146]. For some extractants that are short of cell destructive effects, they can be combined to other chelating agents, antibiotics, hypochlorite, detergents, chaotropes, bases and acids with their own mechanism of action on cell disruption. For instance, bases contribute to membrane lipid saponification, while acids cause membrane pore formation. Solvent extraction has obtained high degrees of efficiency and purity in large-scale production of microalgal pigments. As the basic extraction method, it has been widely combined with other cell disruption methods. However, considering the possible applications in the food industry, the scale application of this technique is limited by toxicity and a residual of solvents, which may adversely affect the edible safety of pigments.

Bead milling (BM) uses high-speed beads made of steel, glass or ceramic by microbiological cell collision. Cell disruption occurs because of shear forces produced due to friction and collision. This method is a simple, fast, and low energy input disruption approach, which is suitable for production in the industry. It causes relatively less damage to intracellular substances, and shows a high disruption efficiency. It has been adopted for determining overall C-PC content in cyanobacteria. For extraction, BM is lack of selectivity, and usually operated with additional solvent. When used for extracting pigments from *S. almeriensis* or *Chlorella* sp., it has been proved to be effective and repeatable [147]. The addition of an appropriate solvent can also facilitate the timely release of thermal energy generated from mechanical action, so as to avoid the damage of pigment structures caused by the temperature rise. 

Freeze-thaw (FT) is usually utilized in the laboratories for disrupting cyanobacteria cells and extract PC, C-PC, or PE, for PBPs are preferable to be handled and preserved at low temperatures [148]. As protein pigments, their denaturation is mainly due to high temperatures, which lead to decreasing amounts of alpha helix. The freezing/thawing for three or four cycles were reported to provide best C-PC extraction purity and yield, and the optimum temperature was around 4 °C [148]. Although FT provides high-purity extracts (0.66–0.87), the repeated cycles consume a lot of energy and time. As a result, this approach is only appropriate in laboratories [148].

#### 6.1.2. Pressurized Systems

High pressure homogenization (HPH) can be regarded as a promising and scalable method, it induces cell disruption by creating mechanical impacts of dramatical turbulence, shear stress, as well as cavitation. When combined with solvent extraction, it has been used on high-quality pigments, especially lipid-soluble ones, from different microalgae. For *Nannochloropsis* sp., the 1000 bar HPH can enhance the bioaccessibility of extracted violoxanthin, antheraxanthin, zeaxanthin, and β-carotene for food products, but decreases their contents due to the sharp raise of temperature [119]. To avoid pigment degradation, an additional cooling system is required. 

Pressurized liquid extraction (PLE) has been used in food processing and was lately evaluated as an extraction method. PLE is usually operated at high temperatures (50–200 °C) and pressures (100–200 bar), which is generally believed to destroy the molecular structure of pigments [149]. However, some studies have successfully extracted carotenoids and chlorophylls with acceptable stability via this method. Its protection of stability and recovery can be verified on *C. vulgaris* for lutein, β-carotene, chlorophyll a/b extraction [120], *D. salina* for β-carotene extraction [121], and *Phormidium* spp. for carotenoids extraction [122]. Deeper investigations are required to establish specific PLE strategies for pigments with different structural characteristics.

Furthermore, a method called continuous pressurized solvent extraction (CPSE) was established with more moderate conditions (generally room temperature at 70 °C) to protect pigments from degradation and has been applied in carotenoids extraction from *Gloeothece* sp. at 60 °C [123]. It also reduces solvent consumption via more effective recirculation.

While a pressurized system does not need cell drying and is easy to achieve scale expansion, it can only be conducted at the low cell concentration (usually 0.01–0.85% *w*/*w*), leading to increased energy consumption. Meanwhile, these methods are not specific enough, because cell debris and additional compounds will be produced into the system, which lead to higher requirements for downstream purification.

#### 6.1.3. Wave-Energy Treatment

Microwave-assisted extraction (MAE) and ultrasound-assisted extraction (UAE) are already investigated for optimization in cell disruption and extraction of high-value compounds from microalgae. Acoustic cavitation is a main driving force of UAE, which increases local pressure and induces thinned cell membrane as well as cell disruption, allowing the penetration of solvent. This method is advantageous for the integral extraction of pigments at low temperature ranges (<70 °C) and has been practiced in β-carotene extraction from *S. platensis* and astaxanthin extraction from *H. pluvialis* [115,126]. It can perform outstanding effects on elevating extraction yield of PBP from *Spirulina* sp., but was also claimed to have adverse effects on PC purity, color and antioxidant activity. 

Regarding efficient disruption of microalgae cells, radiation in MAE can cause heat shock and cell wall degradation. This method requires a much shorter time for thermal treatment, thus lessening the restriction of pigment thermostability [149]. It further achieved a lower temperature when performed under vacuum conditions in a recent study [124]. This method has been practiced on the stable extraction of carotenoids, chlorophylls, PE, PC and APC in low treatment temperatures (around 55 °C) from various microalgae, such as *Scenedesmus* sp. and *Spirulina* sp. [150]. So far, the exploration of MAE for microalgal pigment extraction is still not comprehensive enough.

#### 6.1.4. Enzymatic Extraction

Glucanase, glycosidase, lipase and peptidase have been extensively applied to lyse cells with high specificity [144,151]. These enzymes can disrupt the cell wall and/or membrane, thus leading to the exposure of the intracellular contents to solvent. Enzymatic extraction demands mild reaction conditions and no downstream drying steps and displays a higher extraction rate than any other methods. Specifically, lysozyme can be used for APC obtention from *S. platensis* and guarantee its high quality and purity [117]. The large-scale application of this technology requires the search for enzymes with catalyzing ability to cell disruption and the selection of processing conditions that ensures enzyme activity and pigment stability.

#### 6.1.5. Pulsed Electric Field (PEF)

Electric field technology ash been applied in food processing since the 1970s. Pulsed electric fields (PEF) are utilized to be the appropriate extraction approaches of multiple microalgal pigments by employing a pulse charge onto cell membranes to trigger an electroporation effect [152]. In a study about *C. vulgaris*, PEF displayed an excellent effect on cell disruption without massive carotenoid and chlorophyll inactivation. The process can be refined to perform at 25–30 °C to further increase the extraction effect of temperature-sensitive pigments, especially lutein. In the case of PBP, PEF have been recommended for the extraction of PC from *S. platensis*, *Nostoc commune*, and *Porphyridium cruentum* [128,129,130]. However, it was suggested to be unsuitable for microalgal organisms with a resistant cell wall, such as *Oscillatoria okeni*. PEF can also serve as the pre-treatment of other methods, which has been applied on the first stage of solvent extraction of pigments from *Nannochloropsis* spp. and *H. pluvialis* [131,132]. 

PEF requires low amounts of solvent and has been widely accepted as a “green”, scalable and continuable method for industrial extraction. The parameters such as electric field strength, pulse number and duration should be optimized for different microalgal organisms and desired pigments. For instance, cyanobacteria disruption is energy-consuming for electroporation compared with microalgae because of the membrane components and small size [130].

#### 6.1.6. Novel Methodologies

Some novel methodologies suggested for pigment extraction are mostly adaptations for existing technologies, including laser, flotation, high-voltage electrostatic fields as well as ohmic heating.

Laser has been used for cell disruption in *Nannochloropsis oculata* without destroying pigment structures, but it is difficult for scaling-up into large-scale production [142]. Liquid biphasic flotation is used in combination with the liquid biphasic system as well as solvent sublation to protect pigment stability during extraction. It has been recommended for astaxanthin extraction from *H. pluvialis* [153]. Ohmic heating (OH) and high voltage electrical discharge (HVED) may become options with the highest scalability and economic viability. A continuous electric field with high intensity in HVED can preserve pigment stability and quality. In the extraction of chlorophyll and carotenoids from *N. oculata*, HVED acts as the pre-treatment [143]. OH causes Joule effect via an alternate current, which can accelerate cell breakage and electroporation, while its destruction of pigment structures is less than that of classical methods. 

In conclusion, combinations of both intrinsic and extrinsic influences are responsible for the stability of pigment during the extraction process. From the perspective of extraction technologies, many new advances in methods have been proposed. However, most establishments and the optimization of technologies are chasing a higher yield and recovery to meet the demand of industrial production, which usually comes at the cost of the destruction of pigment structure. So far, liquid–liquid extraction and solvent extraction remain those most common approaches used in carotenoids and chlorophylls extraction. On the other hand, the operating parameters also show an impact on the stability of pigments, in which temperature plays a decisive role in the majority of extraction technologies. UAE leads to great results in PBP extraction yield, but unsuitable high temperatures seriously impact on its stability. Moreover, for food pigments, the potential safety concerns, especially in the methods with organic solvents, cannot be ignored. 

Inherently, pigment stability also relies on the microalgal cell characteristics, pigment properties, and its location in cell. The location of lutein in microalgal cell limits the mass transfer rate, similarly, the robust cell wall of *H. pluvialis* is hard to be broken, requiring more extreme strategies that are responsible for more serious damage of pigment structures. In addition, some pigments have special poor thermostability and pH-stability, or poor tolerance to organic solvents. As protein pigments, proteolysis and denaturation of PBPs happen under unsuitable conditions, which directly damage the structure and physiological activity of pigment molecules. Therefore, more and deeper targeted research is demanded to optimize the obtention strategy for different microalgal pigments, so as to simultaneously achieve higher yield and better product quality, for which the stability of pigments is the most basic premise.

### 6.2. Pigment Stability in Food Processing and Improving Strategies

In food manufacturing, preservation, storage and preparation, faster degradation of natural pigments happens frequently. Compared with extraction, the regulation mechanism of operation conditions on pigment stability in this period is extremely complex, with synergistic or antagonistic effects. Several technologies have been established to overcome the instability issue and guarantee their coloring capability.

Chlorophylls display high susceptibility to oxygen, light, temperature, and pH variations. Based on unfavorable situations, chlorophyll discoloration and decomposition take place immediately to limit their commercial-scale application as food colorants. Substituting unstable magnesium ions by copper or zinc ions can generate blue/green metallo-chlorophylls with high stability, but it is required that metal salt contents should exceed the FDA-established limit contents [154]. Physical encapsulation represents an approach, where a coating material protects and accurately releases sensitive bioactive compounds. Wall materials can protect chlorophylls against light degradation and achieve high storage stability, antioxidant activity and water solubility. Nevertheless, high encapsulation efficiency of food pigments requires the coating material to be biocompatible, biodegradable and low toxicity. While appropriate cooking conditions and food matrices can minimize thermal degradation, using chlorophylls as food colorants remains restricted by their vulnerability to acidic environments (pH 3.5–5.0) and illumination [52], thus more investigations of stable chlorophylls are demanded.

Carotenoids are relatively resistant pigments due to their lipid-soluble properties, but their degradation in processing is still noteworthy. Thermal processing contributes in their structural destruction, depriving the nutritional and organoleptic characteristics of foods. High temperatures during baking, refining, frying and other processes trigger the isomerization as well as later carotenoid decomposition [155]. Carotenoids also have resistance to extreme pH levels; nevertheless, bases and acids can induce trans- and cis-isomerization of certain de-esterifications, rearrangements and double bonds. Therefore, particular attention should be paid to pH control in the processing of food and beverages. For compound juices, carotenoid content experienced a sharp reduction when the pH was adjusted to 7–8 while increased at pH ranges of 3–6 [156]. When exposed to oxygen for long periods, oxidation reactions affect the majority of carotenoids in food products, causing the color fading of products. In the end, exposure to light also triggers the breakdown of carotenoids and generates the loss of their stability and biological function of provitamin A. Foods and beverages containing carotenoids are suggested to be packaged in opaque materials. The existing approach to improve carotenoids stabilization consist of encapsulation. β-carotene needs appropriate wall material and spray-drying conditions. When combined with freeze-drying, spray-drying within microcapsules can prevent the decomposition and enhance its stability in the presence of light, ultraviolet light, high temperatures as well as humidity.

Those major factors that affect PBP stability include pH, temperature, and light. pH has been identified as a major factor affecting PBPs dissociation and aggregation in solution and they should be handled at the best pH level to avoid decomposing. With a pH of about 7.0, the PC stable hexameric form predominates the aggregation, avoiding denaturation and stability decreasing [157]. PE is stable at pH = 4.0–10.0, for its secondary structure adopts the stable conformation (hexameric form) at such pH levels [158]. Maintaining stability at a wide pH promotes its applications into the food industry. PBPs, the protein pigments, are degraded manly because of denaturation. An increasing temperature drives the decrease in a-helix quantity, causing stability loss. Thus, food products containing PBPs are recommended to be handled and preserved at a low temperature, and preferable to be preserved under the lower than ambient temperature because they are susceptible to microbiological decomposition [157]. PBPs are also sensitive to light, among which PC shows degradation after light exposure at the 100 μmol photons/m^2^/s intensity [159]. When exposed to long-term light, it tends to lose chromophores, leading to the loss of color and stability. 

The usage of additives is the simplest way of improving PBP stability, especially thermal stability. This method requires no costly or complicated device. Glucose, sodium chloride and sucrose are the protein-stabilizing agents by covering PC surface to protect its chemical structure and increasing water surface tension [157,160]. Benzoic acid displays antimicrobial and antioxidant activity, with great capability of preserving and increasing PBP stability [161]. However, toxicity and flavor of the heavily used additives must be considered in the food industry. Beet pectin is a suitable material for phycobilins complex formation, and it can protect color while enhancing alcalase, bromelain and papain degradation [162]. Microencapsulation was also proven to be capable for improving thermal stability of PBP and resisting gastric acids. 

Overall, microalgal pigments display susceptibility to oxygen, light, temperature, and pH variations in different degrees, ordinary cooking methods were proven to adversely affect their stability. Unfavorable cooking methods and storage conditions will speed up their degradation and discoloration, leading to the loss of their coloring capability and nutritional value. Further research focusing on the stabilization of natural pigments and their chemical interaction with food matrix is needed. To date, the bottleneck has been addressed via several approaches, which show capability to overcome the instability issue, guarantee their coloring capability and strengthen their incorporation in foods. However, only scarce methods are currently available in the real food industry.

## 7. Economic Analysis of Microalgal Pigments in Foods

Consumers’ proclivity towards nutritive, attractive, and clean-label food products has driven a distinct move of market interest towards natural ingredients, especially within the food industry. Microalgae are one of the major photosynthesizers of naturally derived commercial pigments with abundant availability and maintenance, the growing interest of market in the exploitation of microalgae field has been confirmed in many reports [2]. Countries such as the Germany, USA and China annually generate over 19,000 tons of dry microalgae biomass, which is worthy of around USD 5.7 billion [4]. In terms of major microalgae-extracted pigments, their commercial generation was estimated to be about 10,000, 4000, 1000 and 200 tons/year for *Spirulina*, *Chlorella*, *Dunaliella* and *Haematococcus*, respectively [163]. 

The market value of PBP was predicted to continue increasing rapidly in upcoming years, owing to the increasing demand of natural green and blue shades from food (especially confectionery and beverages), pharmaceutical, nutraceutical and the cosmetics industry [2]. Manufacturers are chasing 100% pure and natural food-grade pigments with antioxidation effects, great nutrient levels as well as vibrant pigments from *Spirulina*, the largest source of PBP. *Spirulina* refers to one of the most worldwide cultured microalgae, which may reach above USD 779 million of marker value by 2026. Meanwhile, remarkable CAGR may grow in the Asia-Pacific region, which provides more opportunities for manufacturers. Among *Spirulina*-pigments, PC reached a marker value of USD 112.3 million in 2018, which is expected to exceed USD 232.9 million by the end of 2025 [2]. PC from *Spirulina* is already used in the food, beverages, and cosmetic industries (such as Bloo Tonic^®^, M&Ms^®^ chocolates and B-blue *Spirulina* drink) with an ever-increasing in quantity demand. When used as food supplements, PC has been produced in liquid and powder form by several companies (e.g., Spirulysat^®^, Electric Sky^®^).

In the case of chlorophylls, based on the report from Value Market Research, it reached a marker value of USD 300 million globally, and may surpass USD 463.7 million by 2025 [2]. As one of its major sources, *Chlorella* may reach the market value of USD 210.15 million by 2024, of which Europe still controls the major market sharing.

Their simple use, good appearance, better stability and increased consumer appeal have contributed to the important position of carotenoids in the global pigment market, especially in America and Europe. The global pigment market produced by >600 organisms’ sources may reach 1.84 billion USD by 2026, which is comprised by 2500–10,000 and 300–3000 USD/kg for astaxanthin and β-carotene, respectively. For β-carotene, its market value outpaced 520 million USD in 2020, which may reach 780 million USD in 2027, of which the β-carotene for dietary supplements accounts for over 21%. In the 1980s, *D. salina* began to be commercially produced by Western Biotechnology and Betatene (Australia) for β-carotene production. The company is currently owned by BASF, the biggest manufacturer of natural β-carotene from *Dunaliella*. To this day, *D. salina* is generated with the annual world-wide yield being about 1200 tons [164,165]. For β-carotene, its global market value may reach USD 618.94 million in 2026, with 3.8% increase in CAGR during 2018–2026 [2]. *H. pluvialis* is produced at an annual amount of above 300 tons in US, India and Israel, and the vast majority of its large-scale culture is for astaxanthin production. In 1994, the AstaReal Group first achieved prosperous astaxanthin manufacturing commercially. A Brazilian company named Ocean Drop sells food and cosmetic products incorporated with astaxanthin. In the end of 2026, the market value of astaxanthin may reach USD 800 million globally. Lutein market surpasses USD 308 million per year and is currently growing [166]. Xanthophylls are also candidate biomolecules, and their application in industrial production can be enhanced by further investigations on their novel biological sources as well as productivity efficiency.

Collectively, consumers’ antipathy against the toxic issues of synthetic food colorants has raised the increase of natural pigments in the food market. As the proportion of natural pigments in the consumer market gradually expanded and partially replaced that of synthetic manufacturers, the demand for microalgal pigments is moving on an upward swing, leading to a sharp raise of market value. Microalgae are now the most economical and eco-friendly source of natural pigments, which will continue gaining more usage in various industrial fields in the next years. Microalgal astaxanthin and β-carotene will elevate in their global sales volume, and will maintain their unassailable position in the global market until at least 2026 [167]. The market value of PBP has also risen at an impressive CAGR in recent years, mostly from the microalgae *Spirulina*. Chlorophylls from microalgae also gain increasing attention in the food industry, and will achieve substantial market value in the next five years. To further expand the market size and economic value of these microalgae pigments, market participants are searching for lower-cost and faster technologies, and more comprehensive regulations are demanded.

## 8. Future Perspective and Conclusive Remarks

It is promising to use microalgae as the candidate sources to obtain value-added biochemicals such as pigments. After decades of research and exploration, microalgal pigments have been proven to have excellent coloring ability and beneficial properties, when used in the food industry, they perform well, have an attractive appearance, various nutritional functions and guaranteed edible safety. 

From the perspective of economic benefits, the market consumption of microalgal pigments has been growing at an impressive CAGR and can be expected to continuosly expand in the next five years, meanwhile consumers’ acceptance and belief in them will also achieve a higher degree. These advantages confirm its wide application range and potential value in diverse markets, and some of them have indeed been successfully produced on an industrial scale. However, inapposite culture and extraction strategy may easily lead to pigment structure alterations or irreversible destruction, impacting the product quality and value. Moreover, the large-scale culture strategies for different microalgae and efficient extraction technologies that ensure product stability have not been fully developed yet. On account of the backwardness of these technologies, the production of most microalgal pigments is still confined at experimental stage, and its commercial industrialization is far from reality. Thus, further explorations are still in need to optimize the existing methods and dig untapped potential technologies, referring to microalgae characteristics, pigment properties and biosynthetic metabolism. In general, microalgae are widely acknowledged to become a prominent and popular source of commercial food pigments in the coming future, with in-depth researches conquering those technological bottlenecks. 

## Figures and Tables

**Table 1:** Microalgal sources, bioactivity and applications of natural pigments.

Pigment	Common Microalgal Source	Bioactivities Known	References
Chlorophylls	*Chlorella* sp. *Monoraphidium dybowskii* *Scenedesmus dimorphus* *Chlamydomonas reinhardtii* *Pavlova lutheri* *Chlorella vulgaris*	• Improving immune system • High antioxidant • Anti-carcinogen	[9,10]
β-Carotene	*Dunaliella salina* *Dunaliella bardawil*	• Anticancer • Antioxidant • Suppression of cholesterol synthesis • Vitamin A activity • Protect skin against sunburn • Prevent coronary artery diseases, fatty liver disease, type-2 diabetes, insulin resistance, age-related macular degeneration, and ultraviolet-induced skin cancer and oral carcinomas	[10,11,12,13,14]
Astaxanthin	*Haematococcus pluvialis* *Chlorella zofingiensis* *Chlorella* sp.	• Antioxidant and photoprotector • Anti-inflammatory, antineoplastic and anticancer • Antimicrobial • Anti-hyperlipidemia, increase serum adiponectin • Beneficial effects on blood rheology and metabolic syndrome	[11,15,16,17,18,19]
Lutein	*Chlorella protothecoides* *Muriellopsis* sp. *Scenedesmus almerienses* *Dunaliella salina* *Dunaliella tertiolecta* *Brassica oleracea* *Spinacia oleracea* *Actinidia deliciosa*	• Antioxidant, prevent the production of free radicals • Immunity strengthens • Anti-inflammatory • Anti-atherogenic and antihypertensive • Cytoprotection against alcohol-induced liver injury • Increase visual sensitivity and filter the harmful blue light • Potent neuroprotection • Prevention of cataracts, age-related macular degeneration, skeletal ischemia, hepatotoxicity and cardiovascular diseases	[10,20,21,22,23]
Fucoxanthin	*Cylindrotheca closterium* *Phaeodactylum tricornutum* *Isochrysis galbana* *Mallomonas* sp. *Nitzschia laevis* *Odontella aurita* *Chaetoceros* sp.	• Stimulate levels of cytokines • Reduce blood triglyceride concentration • Anti-inflammatory, inhibit pro-inflammatory factors, improve phagocytic and microbicidal capacity • Antioxidant, decrease oxidative damage to lipids/proteins • Antineoplastic, inhibit the growth of human leukemia cells and neuroblastoma cells • Anti-obesity	[10,17,24]
Phycobiliproteins	*Spirulina platensis* *Geitlerinema* *Porphyridium* sp.	• Cytotoxicity • Apoptosis • Anti-alzhelmeric activity • Antioxidant	[10,11,12,16]

**Table 2:** Common application of microalgal pigments and their target food products as colorants.

Pigment	Color	Applications	Target Foods
Chlorophylls	Green	• Food ingredients • Food colorant	Beverages, fruit juices, pasta, dairy products, sweetener, preparations, soups
β-Carotene	Red orange	• Food colorant • Feed additive • Vitamin A source	Butter, margarine, cakes, cheese, dairy products, soft drinks, baked products, canned foods, confectioneries, health condiments, dairy products
Astaxanthin	Red orange	• Food colorant • Feed additive • Dietary supplements • Functional foods • Cosmetics	Foods, nutraceuticals, pet foods
Lutein	Yellow orange	• Food colorant • Functional foods	Dairy products, soft drinks, confections, flavorings, salads, pastries, confectionery, infant formula
Fucoxanthin	Yellow brown	• Feed additive • Dietary supplements • Cosmetics	Egg yolk, butter, pie, cakes and other baked foods
Phycocyanin, Allophycocyanin	Blue	• Food colorant • Fluorescent markers	Sweets, ice cream, food, nutraceuticals, chewing gum, jellies
Phycoerythrin	Red	• Food colorant	Sweets, ice cream, food, nutraceuticals, chewing gum, jellies

**Table 3:** The reported extraction methods of microalgal pigments ^a^.

Extraction Method	*Microalgae*	Target Pigments	Solvent	Temperature (℃)	Pressure (Bar)	References
Solvent extraction	*Isochrysis galbana*	Fucoxanthin	ethanol	RT		[35]
	*H. pluvialis*	Astaxanthin	ethanol:ethyl acetate (1:1, *v*:*v*)	RT		[115]
	*S. platensis*	PBP	sodium phosphate	25		[116]
Enzymatic HPH	*A. platensis*	APB	lysozyme + surfactants	37		[117]
	*Nannochloropsis* sp.	Carotenoids	3% *w*/*w* NaCl	n.s.	1380	[118]
	*Chlorella* sp.	Carotenoids	3% *w*/*w* NaCl	n.s.	1070	[118]
	*Tetraselmis* sp.	Carotenoids	3% *w*/*w* NaCl	n.s.	170	[118]
	*Nannochloropsis* sp.	Violoxanthin, antheraxanthin, zeaxanthin, β-carotene	water/recovered with hexane:isopropanol (3:2, *v*:*v*)	n.s.	1000	[119]
PLE	*C. vulgaris*	Lutein	ethanol:water (9:1, *v*:*v*)	148	100	[120]
		β-carotene	ethanol:water (9:1, *v*:*v*)	117	100	[120]
		Chlorophyll a	ethanol:water (9:1, *v*:*v*)	173	100	[120]
		Chlorophyll b	ethanol:water (9:1, *v*:*v*)	170	100	[120]
	*D. salina*	β-carotene	ethanol	160	100	[121]
	*Phormidium* spp.	Carotenoids	ethanol	150	100	[122]
CPSE	*Gloeothece* sp.	Carotenoids	ethanol	60	180	[123]
MAE	*Porphyridium purpureum*	PE	water	40		[124]
		PC/APC	water	100		[124]
	*Cylindrotheca closterium*	Fucoxanthin	acetone	56		[125]
UAE	*A. platensis*	β-carotene	ethanol	30		[115]
	*H. pluvialis*	Carotenoids	heptane	41.1		[126]
PEF	*C. vulgaris*	Carotenoids	citrate- phosphate McIlvaine	n.s.		[127]
	*A. platensis*	C-PC	water	40		[128]
	*Nostoc commune*	C-PC	water	40		[129]
	*Porphyridium cruentum*	PC	citrate-phosphate McIlvaine	20–30		[130]
PEF pre-treatment	*Nannochloropsis* spp.	Carotenoids	recovered in DMSO	20		[131]
	*H. pluvialis*	Astaxanthin	recovered with ethanol	20		[132]
SC-CO_2_	*D. salina*	β-carotene	SC-CO_2_	27.5	443	[133]
	*D. salina*	Carotenoids	SC-CO_2_	30	400	[134]
	*Nannochloropsis oculata*	Carotenoids	SC-CO_2_ + Ethanol	50	350	[135]
	*Nannochloropsis gaditana*	Carotenoids, chlorophylls	SC-CO_2_	60	400	[136]
	*Scenedesmus obliquus*	Carotenoids	SC-CO_2_	60	250	[137]
	*H. pluvialis*	Astaxanthin, lutein	SC-CO_2_	50	550	[138]
	*H* *. pluvialis*	Astaxanthin	SC-CO_2_ + Ethanol	60	300	[139]
	*Synechococcus* sp.	β-carotene	SC-CO_2_	50	358	[140]
	*Synechococcus* sp.	Carotenoids	SC-CO_2_	50	300	[141]
Laser	*N. oculata*	Carotenoids, chlorophyll a	water	n.s		[142]
HVED	*N. oculata*	Chlorophylls, carotenoids	water	20–30		[143]

^a^ Abbreviation: HPH, high pressure homogenization; PLE, pressurized liquid extraction; CPSE, continuous pressurized solvent extraction; MAE: microwave-assisted extraction; UAE, ultrasound-assisted extraction; PEF, pulsed electric fields; SC-CO_2_, supercritical carbon dioxide; HVED, high voltage electrical discharge; RT, room temperature.
